# Supplementary material for: Biomarkers and Predictive Factors for Treatment Response to Tumor Necrosis Factor-α Inhibitors in Patients with Psoriasis
Source: J Clin Med. 2023 Jan 27;12(3):974. doi: 10.3390/jcm12030974 (PMC9918195; doi:10.3390/jcm12030974)
Supplement: Supplementary file 1 [file jcm-12-00974-s001.zip › Supplemental Table 1.pdf]

Supplemental Table S1. The correlations between percent reduction of psoriasis area and severity index (PASI) at week 12 versus age, body mass index (BMI), disease duration, baseline values of PASI or laboratory parameters

	Whole TNF-α inhibitors (55)		IFX (28)		ADA (15)		CZP (12)	
Background factors	*rho*	*p*	*rho*	*p*	*rho*	*p*	*rho*	*p*
Age	-0.202	0.138	-0.166	0.399	-0.388	0.153	-0.0561	0.862
BMI	-0.0308	0.825	0.0707	0.721	-0.486	0.0779	0.063	0.846
Disease duration	-0.207	0.13	-0.231	0.236	-0.204	0.466	-0.342	0.277
Baseline PASI	0.242	0.0751	0.266	0.171	0.182	0.516	0.125	0.699
Baseline CRP	0.136	0.322	0.0861	0.663	0.115	0.684	0.384	0.217
Baseline NLR	0.00791	0.954	-0.178	0.365	0.333	0.226	0.413	0.182
Baseline MLR	-0.158	0.249	-0.167	0.396	-0.143	0.611	-0.179	0.579
Baseline PLR	-0.0337	0.807	-0.178	0.366	0.284	0.304	-0.102	0.753

Correlations between variables were examined using Spearman's correlation coefficients.

TNF-α, tumor necrosis factor-α; IFX, infliximab; ADA, adalimumab; CZP, certolizumab pegol;

NLR, neutrophil to lymphocyte ratio; MLR, monocyte to lymphocyte ratio; PLR, platelet to lymphocyte ratio;

CRP, C-reactive protein
