# Supplementary material for: Biomarkers and Predictive Factors for Treatment Response to Tumor Necrosis Factor-α Inhibitors in Patients with Psoriasis
Source: J Clin Med. 2023 Jan 27;12(3):974. doi: 10.3390/jcm12030974 (PMC9918195; doi:10.3390/jcm12030974)
Supplement: Supplementary file 1 [file jcm-12-00974-s001.zip › Supplemental Table 2.pdf]

Supplemental Table S2. The relationship between percent reduction of psoriasis area and severity index (PASI) at week 12 versus sex, presence or absence of arthritis, scalp, or genital lesions

		Sex			Arthritis			Scalp lesions			Genital lesions		
Biologics (*n*)		Male	Female	p	Absence	Presence	*p*	Absence	Presence	*p*	Absence	Presence	*p*
Whole TNF-α inhibitors (55)†	*n* (%)	43 (78.2)	12 (21.8)		17 (30.9)	38 (69.1)		13 (23.6)	41 (74.5)		31 (56.3)	17 (30.9)	
	Percent reduction of PASI	67 [37-86]	77 [54-82]	0.596	74 [22-80]	71 [50-87]	0.466	77 [48-77]	75 [40-88]	0.511	74 [37-90]	67 [50-78]	0.804
IFX (28)†	*n* (%)	6 (21.4)	22 (78.6)		13 (46.4)	15 (53.6)		7 (25.9)	20 (71.4)		15 (53.6)	8 (28.6)	
	Percent reduction of PASI	76 [28-90]	81 [56-91]	0.37	74 [9-78]	83 [70-91]	0.0557	77 [62-82]	77 [34-91]	0.846	82 [61-91]	69 [43-77]	0.42
ADA (15)‡	*n* (%)	12 (80.0)	3 (20.0)		4 (26.7)	11 (73.3)		4 (26.7)	11 (73.3)		10 (66.7)	3 (20)	
	Percent reduction of PASI	56 [33-71]	79 [67-81]	0.426	76 [58-88]	56 [37-73]	0.327	50 [25-67]	57 [47-84]	0.395	55 [35-79]	67 [49-73]	1
CZP (12)‡	*n* (%)	9 (75.0)	3 (25.0)		0 (0)	12 (100)		2 (16.7)	10 (83.3)		6 (50)	6 (50)	

	Percent reduction of PASI	66.0 ± 2	50.2 ± 27.9	0.397	Not done	61.1 ± 7.9	62.2 ± 29.2	0.96	57.2 ± 36.0	66.9 ± 14.0	0.55

TNF-α, tumor necrosis factor-α; IFX, infliximab; ADA, adalimumab; CZP, certolizumab pegol

†Data are provided as the median [interquartile range], analyzed by Mann-Whitney U test.

‡Data are provided as mean ± standard deviation, analyzed by Student's *t*-test.
