# Supplementary material for: Biomarkers and Predictive Factors for Treatment Response to Tumor Necrosis Factor-α Inhibitors in Patients with Psoriasis
Source: J Clin Med. 2023 Jan 27;12(3):974. doi: 10.3390/jcm12030974 (PMC9918195; doi:10.3390/jcm12030974)
Supplement: Supplementary file 1 [file jcm-12-00974-s001.zip › Supplemental Table 3.pdf]

Supplemental Table S3. The relationship between percent reduction of psoriasis area and severity index (PASI) at week 12 versus presence or absence of nail lesions, diabetes mellitus, current smoking, or bio-switch

		Nail lesions			Diabetes mellitus			Current smoking			Bio-switch		
Biologics (*n*)		Absence	Presence	*p*	Absence	Presence	*p*	Absence	Presence	*p*	Absence	Presence	*p*
Whole TNF-α inhibitors (55) †	*n* (%)	23 (41.8)	30 (54.6)		47 (85.5)	8 (14.6)		14 (25.5)	37 (67.3)		39 (70.9)	16 (29.1)	
	Percent reduction of PASI	75 [53-86]	67 [39-83]	0.435	74 [42-83]	71 [40-91]	0.867	71.0 [45-81]	74 [50-88]	0.712	76 [49-90]	56 [33-80]	0.201
IFX (28) †	*n* (%)	11 (39.3)	15 (53.6)		25 (89.3)	3 (10.7)		8 (28.6)	17 (60.7)		27 (96.4)	1 (3.6)	
	Percent reduction of PASI	77 [61-82]	77 [41-92]	0.755	77 [44-91]	88 [44-94]	0.629	78 [47-82]	77 [63-92]	0.705	77 [41-91]	75 [75-75]	0.804
ADA (15) ‡	*n* (%)	5 (33.3)	10 (66. 7)		11 (73.3)	4 (26.7)		2 (13.3)	12 (80.0)		5 (33.3)	10(66.7)	
	Percent reduction of PASI	67 [56-100]	56 [34-76]	0.269	73 [29-87]	93 [47-97]	0.383	44.44 [38.89-50.00]	62.00 [38.04-80.02]	0.522	67 [54-100]	56 [35-76]	0.462
CZP (12) ‡	*n* (%)	7(58.3)	5(41.7)		11(91.7)	1(8.3)		4(33.3)	8(66.7)		7(58.3)	5(41.7)	

	Percent reduction of PASI	65.2 ± 26.6	57.6 ± 28.8	0.646	57.2 ± 36.0	66.9 ± 14.0	0.55	62.82 ± 30.0	61.64 ± 26.81	0.946	68.79 ± 13.41	52.59 ± 38.38	0.319

TNF-α, tumor necrosis factor-α; IFX, infliximab; ADA, adalimumab; CZP, certolizumab pegol

†Data are provided as the median [interquartile range], analyzed by Mann-Whitney U test.

‡Data are provided as mean ± standard deviation, analyzed by Student's *t*-test.
